# Innovative Strategies for Recruitment in Assisted Living: Lessons From Qualitative and Pilot Intervention Studies

**DOI:** 10.1093/geroni/igaf122.986

**Published:** 2025-12-31

**Authors:** Daniel David

**Affiliations:** New York University, New York, New York, United States

## Abstract

Recruiting assisted living (AL) residents for research presents unique challenges, including resident mistrust, cognitive impairment, and low research engagement. This abstract outlines innovative strategies to recruitment developed through qualitative and pilot intervention studies in three AL facilities. Key to successful recruitment was the Facility-Embedded Nurse Researcher model, where a researcher spent 1 month prior to study initiation to build rapport with staff, residents, and families, enhancing trust and study visibility. This approach established familiarity and interest in the project before the study launch and integrated the principal investigator as part of the residential community. The following aspects will be discussed: 1. Securing support from administration early in the process facilitated recruitment by ensuring frontline staff were informed and engaged with research efforts. 2. Pre-recruitment community events – The investigator led a weekly facility community event to engage residents in discussions about their health needs, goals and values – “Health Talk with Dr. X”. 3. Pre-recruitment facility needs assessment – through staff focus groups, staff identified the need for training on dementia care, palliative care, and resident education on health topic which created reciprocal value and encouraged administrative and staff buy-in. 4. A Community Advisory Board (CAB) helped refine intervention development, recruitment materials, and messaging to align with resident and facility priorities. These approaches improved recruitment efficiency, increased resident participation, and strengthened facility-researcher partnerships. Future studies should integrate these strategies to enhance research feasibility and impact in AL settings.

